# Long-Term Lithium Treatment Increases cPLA_2_ and iPLA_2_ Activity in Cultured Cortical and Hippocampal Neurons

**DOI:** 10.3390/molecules201119663

**Published:** 2015-11-04

**Authors:** Vanessa de Jesus De-Paula, Daniel Shikanai Kerr, Marília Palma Fabiano de Carvalho, Evelin Lisete Schaeffer, Leda Leme Talib, Wagner Farid Gattaz, Orestes Vicente Forlenza

**Affiliations:** Laboratory of Neuroscience (LIM 27), Department and Institute of Psychiatry, Faculty of Medicine, University of Sao Paulo, São Paulo 05403-010, Brazil; vanessajrp@hotmail.com (V.J.D.-P.); dskerr@gmail.com (D.S.K.); mariliapalmafc@gmail.com (M.P.F.C.); schaeffer@usp.br (E.L.S.); leda.talib@gmail.com (L.L.T.); gattaz@usp.br (W.F.J.)

**Keywords:** lithium, neuronal cell culture, iPLA_2_ activity, cPLA_2_ activity

## Abstract

Background: Experimental evidence supports the neuroprotective properties of lithium, with implications for the treatment and prevention of dementia and other neurodegenerative disorders. Lithium modulates critical intracellular pathways related to neurotrophic support, inflammatory response, autophagy and apoptosis. There is additional evidence indicating that lithium may also affect membrane homeostasis. Objective: To investigate the effect of lithium on cytosolic phospholipase A_2_ (PLA_2_) activity, a key player on membrane phospholipid turnover which has been found to be reduced in blood and brain tissue of patients with Alzheimer’s disease (AD). Methods: Primary cultures of cortical and hippocampal neurons were treated for 7 days with different concentrations of lithium chloride (0.02 mM, 0.2 mM and 2 mM). A radio-enzymatic assay was used to determine the total activity of PLA_2_ and two PLA_2_ subtypes: cytosolic calcium-dependent (cPLA_2_); and calcium-independent (iPLA_2_). Results: cPLA_2_ activity increased by 82% (0.02 mM; *p* = 0.05) and 26% (0.2 mM; *p* = 0.04) in cortical neurons and by 61% (0.2 mM; *p* = 0.03) and 57% (2 mM; *p* = 0.04) in hippocampal neurons. iPLA_2_ activity was increased by 7% (0.2 mM; *p* = 0.04) and 13% (2 mM; *p* = 0.05) in cortical neurons and by 141% (0.02 mM; *p* = 0.0198) in hippocampal neurons. Conclusion: long-term lithium treatment increases membrane phospholipid metabolism in neurons through the activation of total, c- and iPLA_2_. This effect is more prominent at sub-therapeutic concentrations of lithium, and the activation of distinct cytosolic PLA_2_ subtypes is tissue specific, *i.e.*, iPLA_2_ in hippocampal neurons, and cPLA_2_ in cortical neurons. Because PLA_2_ activities are reported to be reduced in Alzheimer’s disease (AD) and bipolar disorder (BD), the present findings provide a possible mechanism by which long-term lithium treatment may be useful in the prevention of the disease.

## 1. Introduction

Lithium is a first-line drug for the acute and long-term treatment of bipolar disorder (BD). More recently, evidence derived from experimental models, along with data from epidemiological, neuroimaging and a few clinical studies, has reinforced the potential use of lithium for the treatment and/or prevention of dementia and related neurodegenerative conditions [1]. Lithium has been reported to play a role in neuronal homeostasis [2], stimulation of neuronal growth cones [3], up-regulation of neurotrophins brain-derived neurotrophic factor (BDNF) [4] and vascular endothelial growth factor (VEGF) [5,6], inhibition of glutamatergic excitotoxicity [7], down-regulation of autophagy [8], inhibition of β-amyloid production [9] and toxicity [3], and glycogen synthase kinase 3β-mediated Tau pathology [10].

Early studies suggested that lithium might negatively affect membrane homeostasis through the inhibition of phospholipase A_2_ (PLA_2_) [11], with relevant downstream effects on signal transduction and eicosanoid production [12]. However, a recent study from our group indicates that lithium stimulates hippocampal neurogenesis [13], an effect that apparently depends on the integrity of PLA_2_ function [14]. The independent assessment of distinct PLA_2_ subtypes, including activity and regional distribution, is probably the key to understanding the discrepancies of the enzymatic activity in Alzheimer’s disease (AD) and BD brain.

PLA_2_ is a superfamily of enzymes that are central to membrane phospholipid metabolism and can be divided into three main groups: secretory PLA_2_ (sPLA_2_); calcium-dependent PLA2 (cPLA_2_); and calcium-independent PLA_2_ (iPLA_2_) [15,16,17,18]. These enzymes generally regulate the release of lipid mediators from the cell membrane, playing important roles in signal transduction and regulation of inflammatory response [17,18,19]. Arachidonic acid (AA) is the most important and abundant free fatty acid released from membrane phospholipids through the catalytic activity of PLA_2_ [20,21]. Both c- and iPLA_2_ are highly expressed in the central nervous system, with distinct roles and regional specificities, in addition to a distinct sensitivity and pattern of response to the effect of regulatory cascades. Previous work from our group found that PLA_2_ activity is reduced in AD [22], and that lithium treatment reduces the risk for AD [23]. In the present study, we further develop this hypothesis by analyzing the effect of chronic lithium treatment at different working concentrations (0.02, 0.2 and 2 mM) on PLA_2_ activity in primary cultures of cortical and hippocampal neurons.

## 2. Results and Discussion

**Figure 1:**
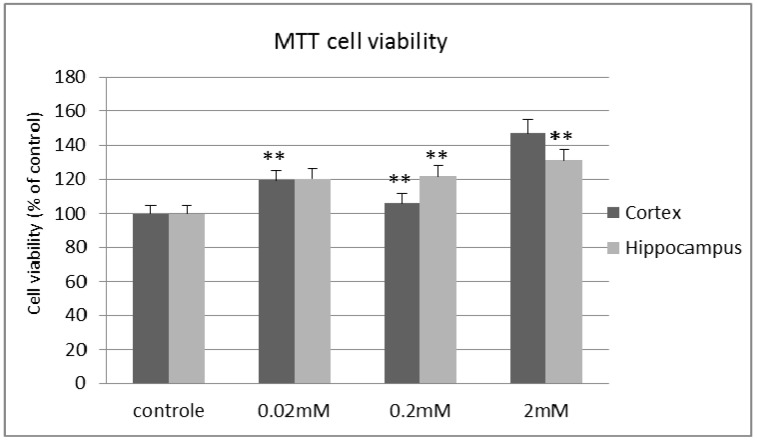
Viabilities of primary cultures of hippocampal and cortical neurons exposed to different concentrations of lithium chloride for 7 days (*n* = 5, ** *p* < 0.01).

The MTT results indicate that treatment with lithium increases neuronal viability in comparison with the control cells, with a marginally significant difference (*p* < 0.05). Samples of cortical neurons show an increase of 19%, 6% (** *p* < 0.01) and 47% in the treatments of 0.02, 0.2 and 2 mM, respectively. The hippocampal cell culture samples show an increase of 20%, 21% and 31% in treatments of 0.02, 0.2 and 2 mM, respectively (Figure 1).

**Figure 2:**
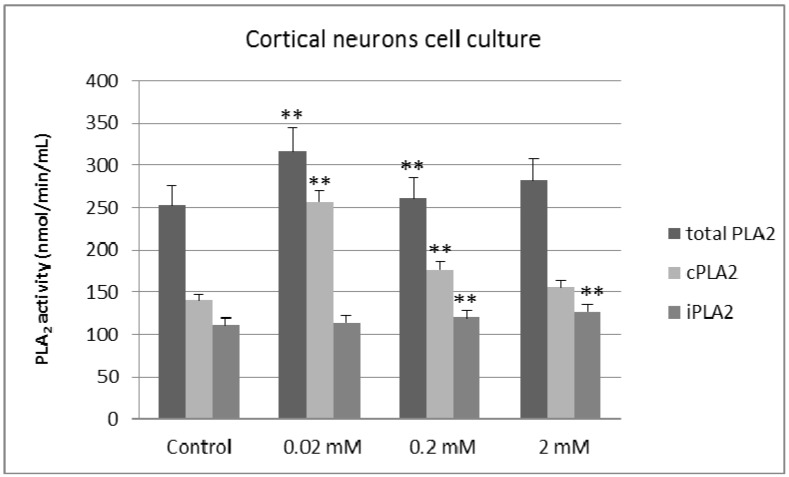
Effects of different concentrations of lithium chloride on the activities of total PLA_2_, cPLA_2_ and iPLA_2_ in cultured cortical neurons (*n* = 5, ** *p* < 0.02).

In cortical neurons, lithium treatment significantly increases total PLA_2_ activity by 25% (0.02 mM; *p* = 0.04) and 3% (0.2 mM; *p* = 0.035), when compared to control. cPLA_2_ activity increases by 82% at 0.02 mM (*p* = 0.05) and 26% at 0.2 mM (*p* = 0.04), when compared to control (Figure 2). Additionally, we found a 7% increase in iPLA_2_ activity at 0.2 mM (*p* = 0.04) and a 13% increase at 2 mM (*p* = 0.05) (Figure 2).

**Figure 3:**
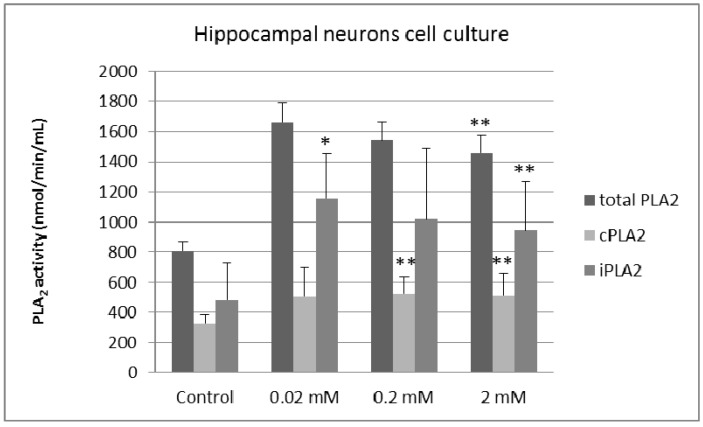
Effects of different concentrations of lithium chloride on the activities of total PLA_2_, cPLA_2_ and iPLA_2_ in cultured hippocampal neurons (*n* = 5, * *p* < 0.05, ** *p* < 0.02).

In hippocampal neurons lithium treatment significantly increased the activity of total PLA_2_ by 80% (2 mM; *p* = 0.04); cPLA_2_ by 61% (0.2 mM; *p* = 0.03) and 57% (2 mM; *p* = 0.04) and iPLA_2_ by 141% (0.02 mM; *p* = 0.0198), 112% (0.2 mM; *p* = 0.2) and 97% (2 mM; *p* = 0.3) (Figure 3).

Our results show that long-term lithium treatment increases both cPLA_2_ and iPLA_2_ activity in primary cultures of cortical and hippocampal neurons at therapeutic and sub-therapeutic concentrations of lithium chloride. This effect was more prominent at micromolar concentrations, with tissue-specific differences in the magnitude of the effect: treatment with 0.02 mM lithium yielded a potent activation of iPLA_2_ in primary cultures of hippocampal neurons, with a 114% increase in enzymatic activity, whereas the same treatment in cortical neurons resulted in a predominant effect on cPLA_2_ (82% increase).

At first sight, the present findings seem to be in disagreement with the results from studies conducted in the late 1990’s, which suggest that lithium might actually inhibit PLA_2_ [11,12]. In the study by Chang and Jones [11] the effect of lithium on total PLA_2_ activity was determined in rat brain homogenates, whilst Chang *et al.* [12] did not assess directly PLA_2_ activity, but made rather an indirect assumption of PLA_2_ inhibition by showing, *in vivo*, that chronic treatment of adult rats with therapeutic doses of lithium decreased the turnover of arachidonate within the total brain contents of phospholipids [7]. The methodological differences between these studies and ours are probably key to the interpretation of the current findings. First, we used a different experimental model (*i.e.*, primary neuronal cultures) to test the hypothesis. Second, we explored this effect using a wider therapeutic window of lithium, *i.e.*, ranging from sub-therapeutic (micromolar) to therapeutic (milimolar) concentrations, which proved to have distinct effects on the target enzymes. Third, we determined the activity of PLA_2_ subtypes using a validated method that specifically reads c- and iPLA_2_ [24]. Finally, we determined PLA_2_ activity in primary cultures predominantly composed by hippocampal or cortical neurons; that is to say, we did not use a total brain model, in which the determination of PLA_2_ activity represents the sum of neuronal and glial PLA_2_, bearing in mind that the enzyme is more constitutively expressed and active in glial cell than in neurons.

Therefore, our data indicate that lithium positively regulates the activity of these two cytosolic forms of PLA_2_. Such an effect may be relevant to the understanding of the trophic effects of lithium, since both c- and iPLA_2_ are implicated in processes related to neurodevelopment and neuroprotection [25]. In the developing human brain, iPLA_2_ is mainly expressed in proliferative zones [26], which have been shown to be sensitive to the neurotrophic effect of lithium [21] including its ability to induce neurogenesis [13].

The reactivity of hippocampal iPLA_2_ to the effect of lithium probably relates to the neurobiological functions of the enzyme in this important cerebral structure. The hippocampus is the brain structure where iPLA_2_ displays its highest documented enzymatic activity [25]. In addition to a well-accepted role in signal transduction through the release of AA and other lipid mediators [27], regulating neurogenesis [26]. Evidence suggests that hippocampal iPLA_2_ is also implicated in the long-term potentiation (LTP) of excitatory synaptic transmission [28,29] and subsequent mechanisms of memory formation [26,28]. Early studies from Clement *et al*. [30] showed that AA was released through PLA_2_ catalytic activity at early stages of LTP induction in membranes prepared from the dentate gyrus. Drapeau and collaborators [31] also proposed that PLA_2_ is involved in hippocampal LTP by increasing the production of AA, which acts as a trophic retrograde synaptic signal to increase transmitter release at glutamatergic synapses [32]. The hypothesis that PLA_2_ facilitates transmitter release in LTP is further supported by the fact that iPLA_2_ participates in membrane fusion, which is a process required for exocytosis [20,33]. Previous findings from our group indicate that the specific inhibition of c- and iPLA_2_ impair neurite outgrowth and decrease the viability of cultured cortical and hippocampal neurons [34]. Accordingly, the inhibition of iPLA_2_ in the rat hippocampus also impairs acquisition of short- and long-term memory [35]. Therefore, one must consider the possibility that the activation of iPLA_2_ in the hippocampus may add to the myriad of neurobiological properties of lithium [36], particularly on the preservation of homeostatic mechanisms related to neuronal response to injury and memory formation [37,38,39].

The stimulatory effect of lithium on cPLA_2_ and iPLA_2_ is particularly interesting in the light of the involvement of PLA_2_ in the pathophysiology of AD. Abnormalities in PLA_2_ have been consistently described in AD patients, showing reduction of enzymatic activity both in the brain regions such as frontal and parietal cortex [40], hippocampus [41] and, peripherally, in platelets of patients with dementia and mild cognitive impairment (MCI) [39,40]. Recent findings from our group indicates that decreased iPLA_2_ activity predicts the risk of conversion from MCI to dementia within the MCI-AD continuum, and decreased cPLA_2_ predicts incident MCI in former cognitively unimpaired elders [37]. Interestingly, these abnormalities seem to respond to treatment with the antidementia drug donepezil, restoring homeostatic levels of enzymatic activity [42]. Therefore, the present findings suggest that lithium treatment may also modify a biological abnormality that is found in patients with, or at risk of AD.

## 3. Experimental Section

### 3.1. Establishment and Treatment of Primary Neuronal Cultures

Pregnant Wistar rats were sacrificed by cervical dislocation at gestational day 18 (E18), and the respective embryos were obtained by laparotomy. Whole embryonic brains were isolated and kept immersed in Hank’s balanced salt solution (HBSS; Gibco, Grand Island, NY, USA). Multiple fragments of cortical and hippocampal tissues were obtained by microdissection, followed by trypsinization (chemical dissociation) and mechanical dissociation. Single-cell suspensions were counted and re-suspended in Neurobasal medium containing B-27 supplement (Gibco), 2 mM glutamine, penicillin (100 I.U.), streptomycin (100 mg/mL), and 5% fetal calf serum (all Gibco). Cells were plated onto poly-d-lysine coated Petri dishes at a density of 1 × 107 cells per culture plate, and incubated for up to 10 days at 37 °C and 5% CO_2_. On day 4 after *in vitro* plated, hippocampal and cortical neurons were incubated for 7 days (37 °C, 5% CO_2_), with different concentrations of lithium (0.02 mM, 0.2 mM and 2 mM). Neuronal viability was microscopically ascertained prior to experimentation. All procedures involving laboratory animals were approved by the Ethics Committee and the Animal Care Committee of the University of São Paulo School of Medicine, in the city of São Paulo, Brazil, and were conducted in accordance with the National Institutes of Health “Guide for the Care and Use of Laboratory Animals” (ISBN 0-309-05377-3, NIH publication No. 86-23, revised 1985; National Research Council 2011).

### 3.2. Assessment of Cell Viability

Cell viability was quantitatively assessed by the MTT (3-(4,5-dimethylthiazol-2-yl)-2,5-diphenyltetrazolium bromide) method, which estimates the percentage of living cells in a given substrate compared to controls [43]. Treatments were replicated at least 5 times with identical experimental conditions. Briefly, cells were plated in the same concentration by surface in a 96 well plate and followed the same treatments described above, after the last day (10 day in culture) of treatment, 50 μL of MTT solution (5 mg/mL in PBS) was added to each well (1 × 10^5^ cells/mL) and the plates were incubated for 3 h, 37 °C and CO_2_ 5%. Then, 500 μL of 10% SDS in 0.01 N HCl was added. After overnight incubation, the absorbance was measured by spectrophotometry at 570 nm.

### 3.3. Determination of PLA_2_ Activity

To determine PLA_2_ activity we used L-α-1 palmitoyl-2-arachidonyl-phosphatidylcholine (Perkin Elmer Life Science, New England Nuclear, Boston, MA, USA) labeled in position 2 with [114C] AA (PC-AA-[114C]) as enzyme substrate. The assay mixture contained 50 μL of 1.0 M Tris-HCl buffer pH 7.5, 200 μL of culture cell homogenates (1 mg of protein homogenate), 150 μL of PC-AA-(114C) (0.12 μCi). For cPLA_2_ measurement we used an optimal concentration of CaCl^2^ (30 μM) and the inhibitor bromoenol-lactone (BEL) to inactivate iPLA_2_ activity (500 μM). Total PLA_2_ was measured with CaCl_2_ 5 mM and vehicle (DMSO). The solution was incubated for 30 min at 37 °C and the reaction was stopped by the addition of 700 μL isopropanol-hydrochloric acid. The ^14^C-labeled AA released by the cleavage of PC-AA-[114C] by PLA_2_ was extracted with *n*-heptane, followed by adsorption of the unbroken phospholipids and the lysophospholipids on 60 mg of silica. The radioactivity of free ^14^C-labeled AA was measured in a Tri Carb Liquid Scintillation counter (Tri-Carb 2100TR: Packard, Meriden, CT, USA). The results were given in CPM (counts per minute) and converted to picomols per milligram of protein per minute using the equation: PLA_2_ activity = CPM × F/A × E × 2.22 × B. The blank counts were subtracted from each sample count (where: CPM = counts per minute; F = adjustment factor for protein concentration; A = specific activity of radioactive substrate in mCi/mmol; B = incubation time in minutes; E = equipment efficiency) [24]. iPLA_2_ activity was inferred by calculating the difference between total PLA_2_ and cPLA_2_ [29].

### 3.4. Statistical Analysis

All experiments were conducted in quintuplicates, yielding mean values for PLA_2_ subtypes (total, c- and i-PLA_2_) in each treatment condition (vehicle or lithium chloride 0.02 mM, 0.2 mM and 2 mM) and model (cortical or hippocampal neurons). Independent sample Student’s *t*-tests were carried out to test for the statistical significance of the difference between mean values of each treatment condition compared to the respective controls. Statistical significance was set at *p* < 0.05. Analyses were performed using the software package SPSS-18 (SPSS Inc., Chicago, IL, USA).

## 4. Conclusions

We provide evidence that long-term lithium treatment activates both forms of cytosolic PLA_2_-cPLA_2_ and iPLA_2_- in primary cultures of cortical and hippocampal neurons. These effects were observed at therapeutic and sub-therapeutic concentrations of lithium chloride, with a more prominent effect at the micromolar range, but definitely without a dose-response pattern. We found that the effect of lithium on these two subtypes of PLA_2_ depends on the brain area from which the primary neurons derive, *i.e.*, iPLA_2_ being more sensitive to the effect of lithium in hippocampal neurons, and cPLA_2_ in cortical neurons. Such differences are probably related to the distinct physiological roles and sensitivity to regulatory mechanisms of c- and iPLA_2_ within the brain. The present findings may be relevant to the understanding of the neurobiological mechanisms of lithium related to neurotrophic response and neuroprotection.
